# Capitalizing on Community Groups to Improve Women’s Resilience to Maternal and Child Health Challenges: Protocol for a Human-Centered Design Study in Tanzania

**DOI:** 10.2196/54323

**Published:** 2024-09-10

**Authors:** Kahabi Ganka Isangula, Aminieli Itaeli Usiri, Eunice Siaity Pallangyo

**Affiliations:** 1 School of Nursing and Midwifery Aga Khan University Dar Es Salaam United Republic of Tanzania

**Keywords:** maternal and child health, maternal and child deaths, human-centered design, income generating associations, sub-Saharan Africa, Tanzania, community groups, community, capitalizing, resilience, maternal deaths, neonatal deaths, mortality, co-design

## Abstract

**Background:**

Maternal and neonatal deaths remain a major public health issue worldwide. Income Generation Associations (IGAs) could form a critical entry point to addressing poverty-related contributors. However, there have been limited practical interventions to leverage the power of IGAs in addressing the challenges associated with maternal care and childcare.

**Objective:**

This study aims to co-design an intervention package with women in IGAs to improve their readiness and resilience to address maternal and child health (MCH) challenges using a human-centered design approach.

**Methods:**

The study will use a qualitative descriptive design with purposefully selected women in IGAs and key MCH stakeholders in the Shinyanga and Arusha Regions of Tanzania. A 4-step adaptation of the human-centered design process will be used involving (1) mapping of IGAs and exploring their activities, level of women’s engagement, and MCH challenges faced; (2) co-designing of the intervention package to address identified MCH challenges or needs considering the perceived acceptability, feasibility, and sustainability; (3) validation of the emerging intervention package through gathering insights of women in IGAs who did not take part in initial steps; and (4) refinement of the intervention package with MCH stakeholders based on the validation findings.

**Results:**

The participants, procedures, and findings of each co-design step will be presented. More specifically, MCH challenges facing women in IGAs, a list of potential solutions proposed, and the emerging prototype will be presented. As of August 2024, we have completed the co-design of the intervention package and are preparing validation. The findings from the validation of the emerging prototype with a new group of women in IGAs and its refinement through multistakeholder engagement will be presented. A final co-designed intervention package with the potential to improve women’s resilience and readiness to handle MCH challenges will be generated.

**Conclusions:**

The emerging intervention package will be discussed given relevant literature on the topic. We believe that subsequent testing and refinement of the package could form the basis for scaling up to broader settings and that the package could then be promoted as one of the key strategies in addressing MCH challenges facing women in low- and middle-income countries.

**International Registered Report Identifier (IRRID):**

DERR1-10.2196/54323

## Introduction

Despite massive improvements in recent years, maternal and child health (MCH) care remains a significant global public health problem. According to the World Health Organization (WHO), 5.2 million children aged younger than 5 years and 295,000 mothers die each year, with low- and middle-income countries accounting for most of these deaths [1,2]. In sub-Saharan Africa (SSA), several countries still face higher maternal mortality rates (MMR) which are persistently above the sustainable development goal target of below 70 deaths per 100,000 live births by 2030 [3,4]. Therefore, most SSA countries are far from achieving the Sustainable Development Goals for maternal mortality [5].

In Tanzania, MMR has continued to be alarming despite some improvements. The Tanzania Demographic and Health Survey and Malaria Indicator Survey have been indicating a fluctuation in MMR. For instance, a decrease from 578 to 454 per 100,000 live births in 2005 and 2010 respectively, followed by a rise to 524 per 100,000 live births in 2017 [6]. Tanzania Demographic and Health Survey and Malaria Indicator Survey reports of 2022 indicate that the neonatal mortality rate has remained steady at 25/1000 live births since the 2015-2016 survey [7]. Amid these fluctuations, the country recently documented an MMR of 362 deaths per 100,000 live births, and an under-5 mortality rate (U5MR) of 56 deaths per 1000 live births [8,9]. Evidence indicates that most of these deaths could have been averted if women had access to high-quality MCH services including expert delivery assistance and postnatal care [10,11]. Therefore, massive efforts are still needed to address the MCH challenges more broadly.

Several community and individual factors significantly influence MCH outcomes, including socioeconomic status, cultural beliefs and practices, health literacy, and social support [12]. These factors collectively contribute to persistently high MMR and U5MR in many regions worldwide [10-12]. Most importantly, socioeconomic status plays a critical role in shaping MCH outcomes. Women and families with lower incomes often encounter formidable barriers to accessing essential health care services. Limited financial resources can impede women’s ability to obtain timely and adequate prenatal care, skilled birth attendance, and postnatal care [12,13]. Consequently, women from economically disadvantaged backgrounds face heightened risks of complications during pregnancy and childbirth, including maternal mortality, preterm birth, and low birth weight [13]. Moreover, negative cultural beliefs and practices prevalent among women of low socioeconomic status also exert significant influence on MCH outcomes by shaping health-seeking behaviors. In economically disadvantaged communities, entrenched norms and taboos may discourage women from attending antenatal visits or using modern contraceptives [14]. This reluctance contributes to higher rates of unintended pregnancies, unsafe abortions, and childbirth complications, exacerbating poor MCH outcomes. Furthermore, inadequate health literacy, often prevalent among mothers and families with low socioeconomic status, further compounds these challenges. A limited understanding of the importance of MCH services and preventive health care practices hinders appropriate care-seeking behaviors [14,15]. Insufficient knowledge about nutrition, immunization, and child development increases the likelihood of suboptimal care and missed opportunities for early interventions, thereby elevating maternal and child morbidity and mortality rates [15]. Additionally, inadequate social support networks among women of low socioeconomic status amplify the difficulties faced in accessing and using MCH services. A lack of emotional, informational, or practical support heightens stress levels during pregnancy and postpartum periods, negatively impacting maternal mental health and overall well-being [15]. Addressing socioeconomic determinants of health is crucial for improving MCH outcomes globally. Effective interventions must prioritize enhancing economic opportunities for women with low socioeconomic status, promoting culturally sensitive health care practices, improving health literacy, and bolstering social support networks. These efforts are essential to ensure equitable access to quality MCH services and to mitigate disparities in MCH outcomes across diverse populations.

The WHO in 2017 developed a framework for raising the standard of MCH care with an emphasis on the need for a holistic approach that addresses health systems, community, and individual factors impacting MCH care [16,17]. A key aspect of this framework is the need for the promotion of community engagement and empowerment as an effective strategy for reducing deaths in MCH care [16,18]. In what appears as partly embracing the WHO framework, Tanzania has taken notable steps to improve MCH service quality and use. At the macro level, the government developed and implemented the 2016-2020 Maternal and Newborn Health Road Map and the 2021-2026 health sector strategic plan, both of which focused on addressing infrastructure and resource limitations, improving health care provider training, and increasing access to essential medicines and supplies [17,19]. At the micro level, the country has implemented the MCH scorecard supported by ALMA2030 and the *Mama na Mtoto* program involving training, mentorship, facility upgrading, and support to health care providers [20,21]. In collaboration with some development partners, the country implemented a community health worker program involving training and deployment of community members to provide basic health care services in their communities, including MCH [22]. Other notable efforts included the m-mama intervention coimplemented by Vodacom Mobile Company, which connects pregnant women in emergency care in rural regions [23].

A major problem with the current interventions for reducing MMR and U5MR in Tanzania is the inadequate engagement of the end users (community members) in the design and implementation and their evaluation. There has been a tendency to adopt a top-down approach in developing MCH interventions posing a risk of not effectively addressing the needs and priorities of local communities. Evidence indicates that community engagement is a key driver of the success of many health care interventions [24,25]. Community engagement is crucial in addressing the deficiencies of MCH care as it helps identify and address the unique needs and challenges of each community [26,27]. Engagement of community members in MCH intervention design, implementation, and evaluation cannot be successful if women are excluded.

Women play an important role in families; therefore, MCH is central to their health. However, they indeed hold less or no power and most decisions on MCH care with financial implications are often fully controlled by men, particularly in low-income countries [28,29]. Nevertheless, socioeconomic transformations are occurring both globally and in SSA where women are becoming more and more supported politically. For example, women are increasingly coming together in Income Generation Associations (IGAs) that are widely supported as one of the efforts to uplift their economy [30]. Tanzania, for example, has a large number of women’s groups including Village Community Banks (VICOBAs), farmers’ groups, and self-help groups. VICOBAs for instance, operate as a form of microfinance institutions, providing small loans and other financial services to their members for self-income generation [31,32]. The VICOBAs are frequently found by women and are intended to economically empower them.

Since poverty is among the key drivers of poor MCH outcomes, VICOBA and other women IGAs that focus on uplifting socioeconomic status could form an important entry point for cushioning poor women toward unfavorable income-related MCH outcomes [33]. The potential contribution of IGAs in improving MCH outcomes has been previously documented [31]. Consequently, the implementation of participatory interventions with women IGAs has been considered an effective strategy for improving MCH outcomes in low-resource settings [31]. However, such evidence is not well established in Tanzania. More specifically, there have been limited MCH interventions leveraging on the power of women IGAs in addressing income-related drivers of poor MCH outcomes in Tanzania. To partly bridge this scholarly and practical gap, we propose a human-centered design (HCD) study to jointly co-design a package of interventions for enhancing women’s resilience and readiness to respond to MCH needs or challenges. HCD is an innovative approach to problem-solving that leverages insights from the end users of new products, services, and experiences to develop best-fit solutions that are rapidly prototyped and iteratively refined [34]. HCD is thought to provide more community satisfaction as well as greater efficiency and cooperation in the process of developing and implementing public health interventions [34]. In addition, compared to conventional problem-solving techniques in the fields of health care and public health, HCD may lead to interventions that are more effective and long-lasting [34]. We seek to engage women from IGA groups in the design and evaluation of interventions alongside the research team and MCH stakeholders. An emerging intervention package through the HCD process is expected to be widely accepted among women and feasible in local contexts in improving their resilience and readiness (income, knowledge, skills, decision-making, engagement, and advocacy) toward addressing MCH challenges. Through testing of the intervention package, if deemed feasible, it could be scaled up to broader settings and promoted as one of the key strategies in addressing MCH challenges facing women in low- and middle-income countries. Therefore, this study aims to co-design an intervention package with women in IGAs to improve their readiness and resilience to address MCH challenges using the HCD approach.

## Methods

### Study Design

**Figure 1:**
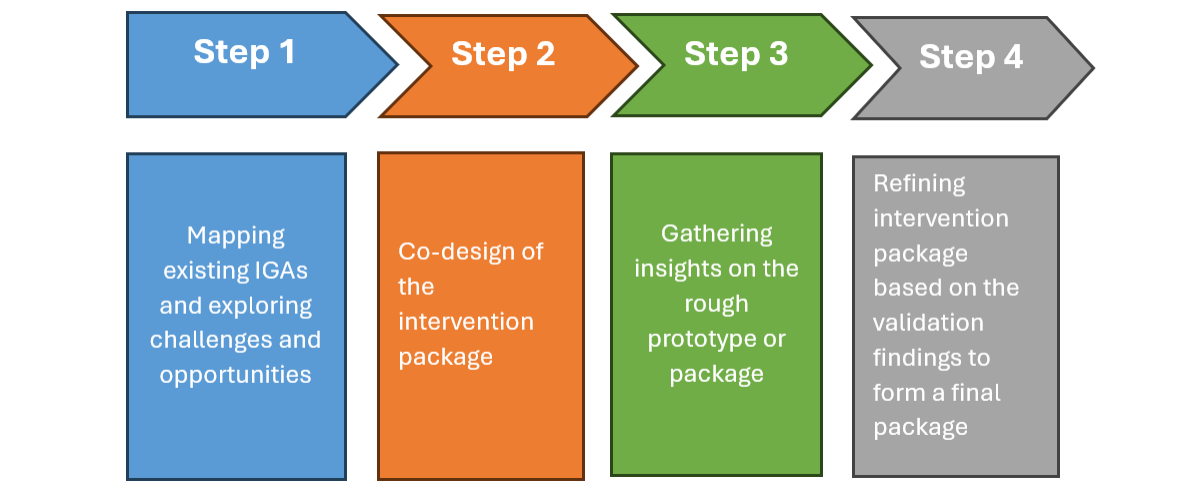
The study’s implementation framework. IGA: Income Generation Association.

The study will use qualitative descriptive design using a 4-step adaptation of the HCD approach as an investigative framework (Figure 1). The use of qualitative descriptive design within the HCD process is aimed at answering the (1) design problem: how can women use IGAs (eg, VICOBA) to improve their resilience and readiness to address MCH challenges? (2) Research question: what are the major challenges facing IGA’s that hinder them from being used to support and help women overcome the MCH challenges? (3) Design question: what is the best intervention package (prototype) co-developed by women in IGAs and stakeholders to address these challenges considering the perceived feasibility, sustainability, and acceptability? The use of a qualitative descriptive approach is also because we aim to develop a deep understanding and describe the challenges and experiences of women in IGA’s (VICOBA). This approach enables a holistic examination of the topic, ensuring that social, cultural, and individual influences are considered and ultimately facilitating the development of targeted intervention using the HCD process.

### Study Setting

The proposed implementation sites for this study are the Shinyanga and Arusha regions in Tanzania. On the one hand, Shinyanga is a region located in the lake Zone forming what used to be Sukuma land. Shinyanga is described in depth by Isangula et al [35]. In a nutshell, the area is predominantly characterized by rural occupancy. The decision to use Shinyanga as a study site was made due to the necessity to draw on the rural experience of Sukuma women, who have less decision-making authority than males, poor MCH indicators, the prevalence of medical pluralism, and the ability to leverage already-existing SONAM networks. Due to its rich IGA groupings, Shinyanga MC was specifically chosen for the research inside the Shinyanga Region. On the other hand, Arusha is home to various ethnic groups, including the Maasai people. The Maasai society is a patriarchal community where men hold the most decision-making power. Given that women in patriarchal communities often have limited access to resources, decision-making power, and social networks, existing community groups can be an essential tool to improve their resilience and readiness to respond to MCH challenges. Within Arusha, Arumeru District is purposefully selected because it has a mix of urban and rural communities, which presents an opportunity to study and explore both urban and rural experiences at the same time.

### Study Population, Sample Size, and Sampling

#### Step 1: Mapping and Community-Driven Discovery Inquiry

This study’s team mapped existing women IGAs in this study’s settings to understand their activities and the level of women’s engagement. This was followed by a qualitative inquiry with the purpose of identifying IGA’s challenges that hinder them from enhancing women’s resilience and readiness toward MCH challenges using a semistructured interview guide. This facilitated the development of an understanding of the local context and the needs of the target population. About 3 focus group discussions (FGDs; in each site) with 6-8 purposefully selected women in IGAs (6 FGDs in total), and 5 key informant interviews (KIIs; in each site) with purposefully selected municipal IGA coordinators and community development officers were conducted (10 KIIs in total). Participants were recruited through IGA coordinators and community development officers. All interviews were conducted at a convenient location confirmed with the respondents in advance to enable them to identify an alternate location if required. Upon arrival, research assistants provided detailed information on this study, obtained informed consent, and engaged respondents for approximately 45 to 60 minutes in a semistructured audio-taped discussion. Data were managed and analyzed thematically using NVivo (Lumivero) software and informed the next step.

#### Step 2: Consultative Co-Design Meetings

Few IGAs’ members, IGA coordinators, and community development officers who participated in FGDs and KIIs were purposefully selected to participate in the co-design meetings. The purpose was to co-design an intervention package aimed at addressing the IGA’s challenges identified in step 1 with a focus on building the resilience and readiness of women toward MCH challenges. A transdisciplinary team of purposefully selected 30 participants (15 women in IGAs and 5 IGA coordinators, 5 community development officers, and 5 other relevant stakeholders) convened for 2 consecutive days. The activities involved (1) a synthesis session to evaluate and validate the qualitative findings from step 1 and share perspectives, experiences, and insights to build a deeper comprehension of the challenges of IGA’s within Arusha and Shinyanga; (2) an ideation session to develop ideas of the potential solutions; and (3) prototyping and co-creation solution**s** to generate the initial (rough) prototype models and components essential to its testing (features, modalities, responsible person, etc). Participants were divided into 3 groups and rated all the proposed solutions on a scale of 0 to 10 for perceived acceptability, feasibility, and sustainability. Commutative scores were computed using Excel (Microsoft Corp) and a moderated discussion was held for the participants to reach a consensus on the top-scoring solutions that formed a rough prototype package. The emerging prototype will guide step 3 of the HCD process.

#### Step 3: Validation and Insight Gathering Inquiry

This step will involve gathering the opinions of women who were not involved in the earlier steps of HCD. The purpose is to gather their insights on the rough prototype considering areas for improvement. We will conduct 3 FGDs with purposefully selected women in IGAs at each site with a focus on separate communities within the selected districts. Women will be recruited through community development officers who are responsible for IGA activities. The FGDs will be conducted at a convenient location confirmed with the respondents in advance to enable them to identify an alternate location if required. Upon arrival, research assistants will provide detailed information on this study, obtain informed consent, and engage respondents for approximately 45 to 60 minutes in a semistructured audio-taped discussion. At the end of the FGDs, participants will also be engaged in rating of the components of the rough prototype from a score of 0 to 10 considering the perceived acceptability, feasibility, and sustainability. FGD data will be managed and analyzed thematically using NVivo software and rating data will be managed and analyzed with Excel. The findings will inform us of the next step.

#### Step 4: Refinement and Adaptation Meeting

The research team will invite 20 MCH stakeholders within Aga Khan University for a 1-day consultative workshop to review the validation findings, refine the intervention package, and form a final package that could be subjected to definitive trials upon securing external grants.

### Data Management and Analysis

Data will be collected by 2 trained research assistants at each site with diplomas in medicine and community development. Data transcription and translation will occur simultaneously by research assistants followed by validation by the coprincipal investigators and the principal investigator. To promote participant anonymity, each interviewee will be assigned a pseudonym, and the transcripts of the interviews will be deidentified. The analytical process will involve a deductive thematic analysis approach [36]. To begin, the research team will collectively review the research questions and, through a consensus-based approach, identify and select various thematic categories. This will lead to the development of an analytical matrix comprising the primary themes and their associated subthemes. Individual transcripts and relevant content representing participants’ responses will then be uploaded into the NVivo (version 12) software for deductive coding. The research team will collaboratively assess any codes that do not align with the predetermined subthemes and themes, removing them if both subjective and objective evaluations indicate that they do not significantly contribute to this study’s objectives. Finally, the research findings will be synthesized into a comprehensive research report by exporting the coded NVivo data into Word (Microsoft Corp), facilitating an interpretative analysis.

### Ethical Considerations

The study has received ethical clearance from the Aga Khan University Ethics Review Committee and was granted a certificate (AKU/2023/08/fb/06/05). Further, this study received approval from the President’s Office–Regional Authority and Local Government and local approvals from the regional medical officers and regional community development officers of Shinyanga and Arusha. We have secured letters of introduction from these entities that will be shared with local community leaders and health care facilities. Before participation, all participants will be asked to provide written consent, which will ensure that this study is conducted ethically. Additionally, the researchers will make sure that there are no repercussions for participants who decide to quit this study at any stage. Limited risks are anticipated because this study does not expose community members or other MCH stakeholders directly or indirectly to any diagnostic activity or treatment. Before analysis, data will be cleaned and potentially identifying information removed. All responses will be kept private as a precaution, and data analysis and reporting will be performed at aggregated levels throughout the Shinyanga and Arusha regions. The collected information will only be used for this research.

## Results

### Participants Demographics

The first section of the results will summarize the participant demographics for each research site considering every HCD step. Participants’ gender, age, primary occupations, marital status, and years of engagement in IGA activities are some of the demographics that will be presented in a tabular form.

### Findings From Community-Driven Discovery Inquiry

The findings from FGDs with women in IGAs and KIIs with stakeholders will be triangulated. Key themes and subthemes related to the challenges faced by women in IGAs and opportunities for addressing them will be examined. Heuristic categorization of themes, subthemes, and relevant categories will be made to ensure a comprehensive presentation of key issues that guided subsequent HCD steps. A focus will be results related to women’s experiences of IGAs including how IGAs operate, perceived benefits, drivers of their desires to join IGAs, challenges facing IGAs, barriers that hinder them from being used to support women overcome MCH challenges, and how women in IGAs respond to these challenges. Findings from health care workers still focus on how they use IGAs to support women in achieving positive MCH outcomes. The findings will be supported by selected participants’ quotes. This section will conclude with how the findings served as a stepping stone for the subsequent HCD steps.

### Findings From the Consultative Meetings

Consultative meetings form a critical step of the co-design process [37]. Participants’ reflections on the findings of community-driven inquiries and suggested refinements as part of the synthesis meetings will be examined. A final categorization of challenges facing IGAs after refinements and reflections will be summarized. Then, a critical analysis and a list of all ideas for the potential solutions to address the identified challenges proposed during an ideation session will be presented. Finally, the findings of prototype and cocreation sessions will be examined. More specifically, rating scores from each participant group for all the potential ideas and cumulative scores will be arranged from the highest to the lowest. The interventions selected to form “a rough prototype model” will be highlighted. The rough prototype may include up to 3 or more interventions with the highest scores. We will use descriptions, tables, figures, and participant quotes for a comprehensive presentation of the findings. It is important to note that, as of August 2024, we have completed the co-design of the intervention package and are preparing validation.

### Findings From the Validation Inquiry

Validation of the rough prototype forms a critical aspect of the co-design process. Ideally, validation could have involved practical testing of the rough prototype in a live setting. However, due to limited resources, we chose to gather insights into women in IGAs who were not included in the initial HCD steps. The findings from FGDs will be synthesized, categorized, and presented. Insights about the features appealing to participants, potential benefits, and modifications or key considerations to improve the implementation success of the rough prototype model will be considered. Individual and summative rating scores of the prototype will be examined and presented in a tabular form. Likewise, the findings will be supported with relevant quotes from the participants.

### Findings From Prototype Refinements or Adaptation Model

The findings from the discussions between the research team and MCH stakeholders will form the key components of the HCD process. A summary of the discussions regarding the validation of the rough prototype and key considerations for the final prototype generation will be developed. The refinements, adaptations, and the final prototype will be presented. The presentation will be accompanied by the figure presenting the final prototype and relevant participants’ quotes.

### Dissemination Plan

Pertinent to the development of a final prototype, the dissemination plan has been devised. The findings of this study will be disseminated through institutional forums (with Aga Khan University) such as university depositories, research meetings, and journal clubs. The summary of the findings in terms of presentations and technical briefs will also be disseminated through national, regional, and district forums to encourage local adoption. We envision disseminating the findings to the international scientific communities through publication in high-impact journals and conference presentations.

## Discussion

### Principal Findings

This study is designed to co-design an intervention package for equipping women in IGAs with essential capabilities to deal with MCH challenges using an iterative process of HCD. The key findings of this inquiry are expected to be 4-fold. First, the finding from the community-driven inquiry with a focus on the potential impacts of IGAs on MCH, women’s engagement practices and challenges hindering IGAs from being used as an entry point to prepare women members to address MCH challenges. The findings will be discussed given previous studies that have examined women’s engagement in IGAs and the potential contribution of IGAs on women, children, and family health [27-33]. Further, the implications of women’s engagement in IGAs on MCH, opportunities for improvements, and barriers that require novel approaches for addressing them will be discussed given previous studies with an emphasis on how HCD forms a promising entry point.

Second are the findings from the co-design sessions with a focus on an emerging rough prototype proposed to address identified challenges. The emerging prototype will be compared with previous interventions for addressing women’s MCH challenges [10-12,17,19-23]. A critical comparison of the potential strengths, weaknesses, opportunities, and threats of the emerging prototype given the previous interventions to improve women’s readiness to handle MCH challenges will be made. We should note that the data management and analysis for this stage are currently in progress.

Third, are the findings from the validation inquiry with a focus on the methodological approach (insight gathering) and adaptations needed to improve the effectiveness of the rough prototype. This step is pivotal to the co-design process. A comparison of our approach (insight gathering) and validation approaches used in previous studies will be made [35,38]. Likewise, the quality of findings of validation through insight gathering in our study will be viewed through the lens of those emerging from previous studies applying similar or different approaches. A methodological question to be answered during the discussion is whether the findings generated through insight gathering suffice amid concerns of limited resources for live rough prototype testing. The section will conclude with how the insights gathered shaped the rough prototype and considerations for future studies on using this strategy when financial resources are limited.

Fourth and finally, are the findings from refinement and adaptation meetings with MCH stakeholders forming the final prototype. Again, a comparison of the methodological approach for the refinement stage used in previous studies will be made. Most importantly, the considerations made by MCH stakeholders to arrive at the final prototype and the refinements made will be discussed and compared with previous literature on interventions for addressing income-related MCH challenges.

### Limitations

To the best of our knowledge, this is the first study to examine the potential value of IGAs in addressing MCH challenges by using the HCD approach, however, it is not without limitations. The first limitation is that some HCD steps used in this study are not similar to the conventional steps described in previous literature [34,35,37,38]. However, this is a recognition of the growing emphasis that HCD need not be prescriptive, rather, steps should be adapted based on expertise, resources, participants, and contexts [38]. Second, we only used VICOBA as exemplars of IGAs, however, we acknowledge the existence of a wide range of IGA groups that could be leveraged to improve women’s readiness to address MCH challenges. While our findings form the basis of exploring this avenue, future inquiries may seek to engage women in groups other than VICOBA. Third and final, while we largely refer to and focus on “women in IGAs,” we acknowledge the role of male partners in MCH care. Exploring male engagement in IGA and whether such engagement could improve women’s readiness and resilience is not a primary focus of this study. However, attempts will be made to draw on men’s perspectives about women’s participation in IGA and the potential contribution to addressing MCH issues, particularly during co-design sessions which are expected to engage male participants.

### Conclusions and Future Recommendations

The HCD approach is an innovative and iterative approach to advance global health and holds a promising opportunity for addressing MCH challenges through the engagement of women in the co-design of effective, sustainable, and acceptable interventions. The study presents an opportunity to apply the HCD approach in designing interventions for addressing income-related barriers to MCH care among women capitalizing on their engagement in IGAs. By actively involving women from IGAs and key stakeholders, we aim to develop an intervention package that not only empowers women economically but also equips them with the resilience and readiness to address MCH barriers effectively. The results of this study have the potential to inform future MCH interventions in low-resource settings and contribute to the global efforts to reduce MMR and U5MR.

## Abbreviations

FGD: focus group discussion
HCD: human-centered design
IGA: Income Generation Association
MCH: Maternal and Child Health
MMR: maternal mortality rate
SSA: sub-Saharan Africa
U5MR: under-5 mortality rate
VICOBA: Village Community Bank
WHO: World Health Organization

## Data Availability

Our HCD intervention package findings will be shared in future publications, ensuring data transparency. However, for ethical reasons and to protect participant privacy, some details may not be disclosed. Additional data requests can be made through the corresponding author [KGI] at the Aga Khan University.
